# Rehabilitation of Visually Impaired People: Experiences and Relevance According to the Perception of Family Members and Patients

**DOI:** 10.3390/healthcare13070712

**Published:** 2025-03-24

**Authors:** Ana Cláudia Fernandes, Yara de Moraes, Pedro Henrique Silva Carvalho, Rita de Cassia Ietto Montilha, Florencia Carmine, Cristian Sandoval, Síbila Floriano Landim, Nicolás Márquez Álvarez, Dolors Rodriguez-Martin

**Affiliations:** 1 School of Medical Sciences, University of Campinas, Campinas 13083-887, Brazil; pesquisa@draanaclaudiafernandes.com (A.C.F.); ydemoraes3@gmail.com (Y.d.M.); p185738@dac.unicamp.br (P.H.S.C.); rcietto@unicamp.br (R.d.C.I.M.); 2 Carrera de Medicina, Facultad de Medicina, Universidad de La Frontera, Temuco 4811230, Chile; f.carmine02@ufromail.cl; 3 Escuela de Tecnología Médica, Facultad de Salud, Universidad Santo Tomás, Osorno 5310431, Chile; 4 Departamento de Medicina Interna, Facultad de Medicina, Universidad de La Frontera, Temuco 4811230, Chile; 5 Núcleo Científico y Tecnológico en Biorecursos (BIOREN), Universidad de La Frontera, Temuco 4811230, Chile; 6 School of Occupational Therapy, Faculty of Psychology, Universidad de Talca, Talca 3465548, Chile; 7 Graduate Program in Health Promotion, Cesumar University (UniCesumar), Maringá 87050-900, Brazil; 8 Escuela de Ingeniería Comercial, Facultad de Economía y Negocios, Universidad Santo Tomás, Talca 3460000, Chile; nmarquez4@santotomas.cl; 9 Department of Fundamental and Clinical Nursing, Faculty of Nursing, University of Barcelona, 08007 Barcelona, Spain; dolorsrodriguezmart@ub.edu

**Keywords:** family, interdisciplinarity, rehabilitation, visual impairments

## Abstract

**Background**: This study aims to explore the perceptions of visually impaired patients and their families regarding rehabilitation. **Methods**: A qualitative, descriptive, and cross-sectional design was employed, incorporating data from semi-structured interviews conducted with patients and their families as part of a broader project. Data were analyzed using a content analysis approach to address the study’s objectives. **Results**: The findings identified three primary categories based on interviews with nine patients and six family members: (A) the impact of rehabilitation on autonomy and identity formation; (B) the significance of professional training, experience, and assistive technology in rehabilitation; and (C) the effects of rehabilitation on patients and their families. **Conclusions**: Patients and their families deemed rehabilitation essential for fostering autonomy and independence in daily activities. A multidisciplinary approach, emphasizing professional training that integrates health, rehabilitation, and education, was found to be vital.

## 1. Introduction

Visual impairment (VI) refers to a congenital or acquired organic deficiency in the functioning of the visual system [1,2]. The primary causes of VI include congenital glaucoma, cataracts, retinopathy, and other conditions [1,2]. The International Classification of Functioning, Disability, and Health (ICF) defines disability based on functionality, representing the dynamic interaction between a person’s health condition, environmental factors, and personal factors. Conversely, the International Classification of Diseases (ICD-11) focuses solely on visual acuity, considering only the body structure and etiology of the condition, and classifies VI into degrees such as mild, moderate, severe, or blindness [1,2,3].

It is important to highlight that the ICF and ICD are complementary WHO reference classifications and are members of the WHO Family of International Classifications. These classifications are not mutually exclusive. In the processes of evaluation, diagnosis, and classification, individual experiences with VI must be considered, as many factors influence the functionality of vision. The ICF aims to assess how vision is used in daily activities [4], supporting treatment and rehabilitation plans that promote social inclusion and accessibility.

Recent estimates suggest that approximately 2.2 billion people globally experience some form of vision impairment or blindness [2]. In Europe, around 25 million individuals are affected by VI, with approximately 979,200 people in Spain living with some form of VI, primarily due to glaucoma and diabetic retinopathy [5].

In Brazil, the most recent census found that 23.92% of the population has some type of disability, 18.6% of whom have VI. Notably, WHO estimates that 80% of VI cases are preventable. With a population exceeding 200 million, this indicates a significant prevalence of VI in the country [1,6]. In the USA, over 1 million individuals are blind, and more than 11 million have low vision, with 8 million cases attributed to uncorrected refractive errors [7].

Although VI is biologically measurable, its impacts extend to various life aspects, including learning, education, daily activities, and professional life. Silva and Camargo [8] assert that these challenges stem not from the disability itself but from environmental factors—both physical and social—that prioritize vision.

This aligns with the “inclusion paradigm” introduced by the Convention on the Rights of Persons with Disabilities [9], which Brazil adopted in 2009 through Decree 6949 and subsequent regulations [10].

In Brazil, Ordinance N°. 793, enacted in 2012, established guidelines for implementing and operating the Care Network for People with Disabilities under the Unified Health System [11]. This network includes components functioning cohesively to ensure comprehensive care and equitable access to health services, such as primary care; specialization in auditory, physical, intellectual, and visual rehabilitation; and emergency/hospital services [11].

Since this ordinance [11], Brazil’s Ministry of Health has actively expanded rehabilitation services by funding new services and enhancing existing ones. Given that habilitation and rehabilitation processes empower individuals with VI by fostering autonomy and training them to use optical and non-optical assistive technology (AT) resources, this investment has significantly benefited the population. These advancements have also created new opportunities for education and daily activities in less inclusive environments, enabling individuals with VI to reach their full potential [12,13].

Assistive technology encompasses resources, systems, services, and methodologies aimed at enhancing functionality and independence, thereby improving quality of life. According to the WHO, “without assistive technology, people are often excluded, isolated, and locked into poverty, thereby increasing the impact of disease and disability on individuals, their families, and society” [14].

The family plays a crucial role in the rehabilitation of individuals with VI. Othero & Ayres [15] emphasize that family, friends, and health professionals in rehabilitation institutions form a supportive network aiding patient decision-making. Additionally, families should be integral to the rehabilitation team, participating in therapeutic approaches and decision-making processes. Developing trust between families and professionals fosters humanized care through equitable relationships [16].

The way a diagnosis is communicated to families significantly impacts the rehabilitation process. Authors such as Figueiredo et al. [17], Fernandes & Montilha [18], and Fernandes et al. [19] emphasize the importance of interventions that address family concerns, clarify medical diagnoses, and provide appropriate care for individuals with VI.

Furthermore, given that many caregivers must forgo work and other activities to support rehabilitation, assessing the familial impact is critical. Focusing on families and users helps to understand their experiences, from diagnosis to rehabilitation, and to optimize the resources available within health networks [20]. Understanding patient and family perceptions of rehabilitation is vital for identifying opportunities to improve services [21].

This study aims to explore the perceptions of individuals with VI and their families regarding rehabilitation services. It investigates the impact of these services on autonomy, identity, and quality of life, identifying deficiencies and opportunities for enhancement to optimize support for this demographic.

## 2. Materials and Methods

This study adopts a qualitative, descriptive approach, utilizing semi-structured interviews conducted in a municipality within the metropolitan region of Campinas, São Paulo, Brazil, in 2018. Qualitative research emphasizes understanding meanings, experiences, and perspectives. The study is grounded in a phenomenological paradigm, aiming to explore the lived experiences of visually impaired individuals and their families. Phenomenology facilitates a deeper comprehension of the perceptions and meanings [22] associated with the rehabilitation process by focusing on individual narratives within their contextual frameworks. These narratives, while subject to change over time, often reflect enduring values, convictions, and cultural practices [23].

To ensure the reliability and effectiveness of the interview process, a pre-test was conducted to refine the questionnaire and enhance its validity as a data collection instrument. The questionnaire included questions about participants’ demographic profiles, the diagnostic process, the professionals involved in initial consultations and subsequent rehabilitation, and the healthcare and rehabilitation professionals’ roles. Additionally, the questionnaire sought to identify areas for improvement within the healthcare and rehabilitation network [24].

### 2.1. Ethical Considerations

This study, titled “Challenges and Potentialities of Visually Impaired People’s Therapeutic Itinerary: Discovering Warps and Connections”, is part of a broader Ph.D. thesis research project approved by the Research Ethics Committee of the University of Campinas under CAAE No. 46001215.7.0000.5404. The project is entitled “The itinerary of people with visual impairment in healthcare and rehabilitation services in a municipality in the Metropolitan Region of Campinas/SP, Brazil”.

The study adhered to the ethical guidelines for research involving human subjects, as stipulated by Resolution CNS/MS-466/12 (Brazil) and complementary regulations. These regulations guided the preparation of instruments and the informed consent process. Additionally, the research followed the bioethical principles outlined in the Belmont Report [25] and the Declaration of Helsinki [26].

### 2.2. Participants

The participants and the investigation field were selected based on feasibility and convenience. The inclusion criteria ensured that participants had a direct connection with the rehabilitation services being studied, including patients from the municipality’s referral rehabilitation center, their family members, and local residents. There were no exclusion criteria regarding sex, age, or type of eye condition, as the study aimed to capture diverse experiences and perceptions of rehabilitation.

All participants were fully informed about the study objectives and provided written consent by signing an informed consent form.

The study included nine users (four adults and five children) (Table 1), identified as “P” (patient) followed by a number (P1–P9). Additionally, six family members participated: four mothers, an aunt, and a husband. Family members were labeled “FM” (family member) to maintain participant confidentiality. For underage users, their respective family members answered the interview questions.

In one instance, the guardian of an adult participant (P3) answered the questionnaire because the patient was unable to respond due to a combination of non-literacy and communicative restrictions caused by their disability. Patients P4 and P5 were siblings; their mother contributed to the study by sharing distinct experiences for each child. Participant P9 participated in the interview with her husband, who regularly accompanies her to health and rehabilitation services. Both authorized the use of their responses to enrich the study.

The sample size of nine patients and six family members aligns with the qualitative nature of the research, which prioritizes an in-depth understanding of participants’ experiences and perceptions rather than generalizing results to a broader population [27]. Qualitative research does not require large sample sizes to achieve data saturation. Prior studies, such as the one conducted by Guest et al. [28], suggest that saturation can be reached with a relatively small number of interviews, depending on the sample’s homogeneity and the richness of the collected data.

Systematic analysis of the data revealed that participants’ responses reflected similar experiences and perceptions, indicating that the study achieved a comprehensive understanding of the phenomenon under investigation [29].

**Table 1:** Characterization of visually impaired patients (prepared by the authors).

Patients	Age	Gender	Educational Level	Visual Impairment	Related Cause	Family Member	Relationship
P1	69	M	Higher education	Acquired blindness	Pigmentary Retinosis	*	*
P2	29	M	Undergraduate	Acquired low vision	Traumatic Neuritis	*	*
P3	22	M	Illiterate	Congenital blindness	Toxoplasmosis during pregnancy	FM3	Mother
P4	6	M	Middle School	Congenital low vision	Albinism	FM4/5	Mother
P5	7	F	Middle School	Congenital low vision	Albinism	FM4/5	Mother
P6	6	F	Middle School	Congenital low vision	Trauma during pregnancy	FM6	Mother
P7	5	M	Preschool	Congenital low vision	Nystagmus and Photophobia	FM7	Aunt
P8	9	M	Middle School	Acquired low vision	Retinal detachment	FM8	Mother
P9	25	F	High School	Acquired low vision	Stargardt’s disease and Best’s disease	FM9	Husband

* Family member data is unavailable for these cases, as they did not participate in the study.

### 2.3. Data Analysis and Methodological Rigor

The data used in this study are part of a broader research project and were analyzed using a content analysis approach, which involves reading, coding, and interpreting the content in a systematic and refined manner [30]. Initially, a thorough review of the interview transcripts was conducted to familiarize the researchers with the data and to gain insight into the participants’ experiences and perceptions. During this phase, recurring themes and emerging patterns were identified [30].

Subsequently, a coding process was undertaken, during which labels or codes were assigned to specific segments of text that represented relevant concepts or categories [31]. This step facilitated the organization of data in a structured way, enabling more detailed analysis.

Finally, the content interpretation phase focused on analyzing the identified codes and categories to extract latent meanings and uncover relationships among them. This interpretative process was conducted with precision, taking into account the context of the responses and the interconnections between different themes [30].

By integrating these analytical techniques, the study was able to extract collective insights regarding participants’ experiences, opinions, messages, and perceptions, thereby enabling a comprehensive understanding of the phenomenon under investigation [28].

## 3. Results

Three categories of analysis as shown in tables, were developed based on the data collected, as follows: A—impact of rehabilitation on the autonomy and constitution of the subject’s identity; B—relevance of professional training and experience, assistive technology resources and rehabilitation; and C—impacts of rehabilitation on patients and family members.

### 3.1. Category A. Impact of Rehabilitation on the Autonomy and Independence of Persons with Visual Impairment

This category of study will examine the impact of the rehabilitation experience on the participants’ ability to function independently, as well as its influence on their self-perception and sense of identity (Table 2).

**Table 2:** Category A—Impact of rehabilitation on the autonomy and independence of persons with visual impairment.

Patient	Age	Statements
P1	69	“I’ve already taken a bus here at the terminal and went to São Paulo to meet my son… In my opinion, you feel much more comfortable when you have knowledge and resources, as it gives me more confidence.”
P2	29	“Sports activities are the most beneficial activities in rehabilitation. I played goalball, which helped me a lot… to learn to control my hearing and sense of space (…), in addition, swimming helped me improve my balance and direction; while kayaking and sailing helped me improve my perception of wind, which helps me know where I am. In my opinion, sports activities are the best part of rehabilitation.”
FM3 (Family Member)	-	“My son would be in a wheelchair and maybe wouldn’t be able to speak or walk if there was no rehabilitation, so I can’t explain, but I believe that rehabilitation is essential to any disability.”
FM6 (Family Member)	-	“She made new friends when she started going to rehabilitation… Now, it is all normal to her and I believe it’s important to people with this condition to have contact with people who also have it, so they can see it is normal… On the other hand, she may feel excluded in a place where she’s the only one with this condition.”
FM7 (Family Member)	-	“I believe that there should be a follow-up at the school, since it’s really, really hard to get the student to understand why he can’t see… They can ask themselves ’Why am I being excluded’, right?”

The participants reported significant improvements in their autonomy and social integration after rehabilitation. This is evident in statements about increased confidence in daily activities (e.g., taking the bus), enhanced social connections, and the ability to perform previously difficult tasks. However, some participants pointed out challenges related to exclusion, particularly in educational settings where peers may struggle to understand the condition. Sports activities were highlighted as particularly beneficial for improving spatial awareness, balance, and overall well-being.

### 3.2. Category B. Relevance of Professional Training and Experience, Assistive Technology Resources, and Rehabilitation

This category explores the relationship between the level of training professionals possess and their access to specific technologies, and the effectiveness of the rehabilitation process (Table 3).

**Table 3:** Category B—Relevance of professional training and experience, assistive technology resources, and rehabilitation.

Patient	Age	Statements
P1	69	“I had a very interesting experience with computing (…), since I had a blind teacher who had exactly the same condition as I have. And without discrediting them, but this is different from the professionals here… It’s just an analogy, since I had both experiences. A teacher who has the same condition relates to you on a deeper level, as he understands what you are going through, but that is not to say the professionals here are any less committed.”
P2	29	“In my opinion, rehabilitation is especially important… I liked it at the beginning, but then… I mean… I was taking the computer training course, then a new student was introduced; then we had to review everything up to that point. So, if the student is absent, I can’t move on for the day… I believe that this doesn’t really help.”
P9	25	“I like rehabilitation because it is a place where I learn things and I’m fully supported by professionals who understand the disability… as they are not included in society, people affected by this condition want to isolate themselves, and rehabilitation helps in this matter… It is very important to have some guidance.”
FM7 (Family Member)	-	“There is a great support… whoever finds that place is well-served, the equipment are particularly good, the doctors are very attentive, and the rehabilitation is very good…”
FM9 (Family Member)	-	“The health professional present during screening is not aware that she has low vision and lets her bump on things and sit on the floor.”

The relevance of professional training and access to assistive technology was highlighted by most participants. Some emphasized the importance of interacting with professionals with direct experience of the condition, as they were able to relate on a deeper level. However, there were also critiques about the lack of understanding and preparation of some professionals, which impacted the quality of care. Assistive technology and structured rehabilitation programs were recognized for their value in improving daily living skills and social inclusion.

### 3.3. Category C. Impacts of Rehabilitation on Patients and Family Members

This category delineates the alterations undergone by patients and their families in the course of rehabilitation processes. This category explores the impact of rehabilitation on patients’ quality of life and family dynamics (Table 4).

**Table 4:** Category C—Impacts of rehabilitation on patients and family members.

Patient	Age	Statements
P2	29	“To be honest, I went there only twice… It wouldn’t really make a difference, so I said, ’it’s not worth it for me’ to leave my city just to learn Braille, which is something that I do not use… And I learn how to use the computer by using it, and I barely even use a cane, only at night, for that I took classes at [name of rehabilitation service].”
FM5 (Family Member)	-	“So much has changed: the colors, objects, and they are even talking more. They used to be kind of shy… all of that, they developed a lot. They do pedagogical activities there, with different materials, this and that… The children learn in a playful way, and I believe it had a positive effect.”
FM6 (Family Member)	-	“Since the staff helped her to read and write she improved in many ways. Her first-grade teacher said she did it easily when she was approved.”
FM7 (Family Member)	-	“Yes, I believe it’s great. But the parents also must help at home… we can’t just leave it to the professionals… she’s taught how to do something… Then, when he goes back there in 15 days, he has already forgotten all about it. So, we come up with activities for him to do here, like a toy for him to assemble.”

Rehabilitation positively affected not only the patients but also their family members. The family members noted improvements in communication, social skills, and independence. However, there was an emphasis on the need for continuous involvement from the family, as some patients tended to forget skills learned during rehabilitation sessions without reinforcement at home.

## 4. Discussion

Our results demonstrate the significant role of professional training and assistive technologies in the rehabilitation process. Participants appreciated professionals with lived experiences of visual impairments, as they were better able to address patients’ needs. However, some gaps in training and understanding among healthcare providers and educators were noted, which can undermine the effectiveness of rehabilitation and restrict access to essential services, as FM9 emphasized.

Rehabilitation also brought about positive changes for families, enhancing communication, independence, and social skills. Family members stressed the need for active involvement at home to sustain the benefits of rehabilitation, reflecting the importance of family participation in the process.

Nevertheless, structural barriers persist, particularly in educational settings, where inadequate accommodations for students with visual impairments foster feelings of exclusion. As noted by FM7 and FM6, addressing these challenges requires greater societal commitment to inclusion, extending beyond the rehabilitation context.

Rehabilitation is instrumental in addressing functional challenges and fostering autonomy, as emphasized by Germano et al. [32], particularly among school-age children with visual impairments. However, barriers such as limited access to assistive technologies and societal exclusion persist, significantly hindering the integration and independence of individuals with visual impairments [33]. FM6 and FM7 highlighted the lack of recognition in environments beyond rehabilitation, underscoring the need for broader societal engagement to facilitate inclusion [33].

Children with visual impairments have the potential to establish meaningful relationships and acquire knowledge when provided with adequate opportunities. Nevertheless, as FM7 observed, deficiencies in school settings often exacerbate feelings of exclusion. Coelho and Abreu [34] further identified structural barriers in educational institutions, including inadequate infrastructure and restrictive enrollment policies. These limitations frequently isolate students with visual impairments, reinforcing their identity as “the disabled child” and fostering dependency on teachers [34,35]. Luque et al. [36] contrasted two educational approaches: one focused on “normalization”, which lacks specialized support, and another emphasizing tailored teaching methods that respect the unique needs and perspectives of students with impairments.

Teachers’ attitudes, often shaped by limited understanding of visual impairments, may inadvertently reinforce overprotection, thereby constraining opportunities for growth and independence [34,35]. Botha and Watermeyer [37] expanded on this issue, exploring how ableism—a structural phenomenon—perpetuates stereotypes that frame individuals with visual impairments as dependent or “unfortunate” [38]. Their findings highlight the urgent need to shift narratives and prioritize autonomy through inclusive practices.

The interdisciplinary collaboration of health and education professionals is critical for fostering inclusive interactions and promoting autonomy. As emphasized in the Global Eye Health Action Plan, rehabilitation programs should integrate functional vision assessments, assistive technology, and tailored interventions to address the diverse needs of individuals with visual impairments [39,40]. Physical educators, physiotherapists, and occupational therapists play a pivotal role in enhancing independence through physical activities, which have been shown to improve balance and proprioception, as demonstrated by Corazza et al. [41].

Despite its benefits, the effectiveness of rehabilitation is often impeded by professionals’ limited understanding of visual impairments, as noted by FM9. This lack of knowledge impacts not only the quality of care but also access to essential services, including education and transportation. Blackstone et al. [42] highlighted gaps in Orientation and Mobility programs, which remain inadequately addressed despite other professional support. Targeted training for healthcare and education professionals is essential to equip them with the skills necessary for delivering inclusive, person-centered care [43].

Rehabilitation also yields significant benefits for families, offering emotional support and enhancing their engagement with educational and healthcare services. FM5 emphasized the positive impact on children’s school performance and communication skills, fostering an anti-ableism culture by promoting self-awareness and the recognition of individual potential. However, as Rainey et al. [44] and Fernandes & Montilha [18] observed, families often grapple with the balance between fostering independence and maintaining protective behaviors, particularly during adolescence when concerns about social isolation and future transitions become more pronounced.

The interdisciplinary rehabilitation team, as outlined in Brazilian guidelines, plays a fundamental role in addressing the multifaceted needs of individuals with visual impairments. This team includes contributions from pedagogues, speech therapists, and typhlotechnology technicians, who support autonomy and academic performance, language development, and social interaction [18,43,45,46,47]. Furthermore, the active involvement of families, as emphasized by Fernandes & Montilha [18] and Fernandes et al. [19], significantly enhances rehabilitation outcomes by fostering a collaborative environment that strengthens inclusion and empowers all stakeholders as agents of change.

### 4.1. Limitations of the Study

While this study provides valuable insights into the rehabilitation experiences of individuals with visual impairments, several limitations must be acknowledged. First, the reliance on self-reported data introduces potential bias, as participants’ accounts may not fully or accurately reflect their experiences. Additionally, the study was conducted within a specific geographical region, which restricts the generalizability of the findings to other contexts or countries with distinct healthcare and educational systems.

The study did not include structured interviews or questionnaires tailored to specific age groups, which could have provided a more nuanced understanding of how rehabilitation experiences vary across different life stages. Furthermore, the exclusion of key stakeholders, such as teachers and rehabilitation professionals, resulted in a lack of comprehensive data, which could have enriched the findings by offering a more holistic understanding of the rehabilitation process.

Another limitation is the relatively small sample size of nine patients and six family members. While this sample size aligns with the goals of qualitative research, it inherently limits the generalizability of the findings. Moreover, the inclusion of participants from diverse age ranges, from children to adults, enriched the study by capturing varied perspectives but may have introduced variability that complicates the interpretation of shared themes. These limitations underscore the need for future research including larger, age-specific samples and the perspectives of additional stakeholders to validate and expand these findings.

### 4.2. Directions and Recommendations for Future Research

Future research should develop more structured interviews or questionnaires for patients and their families to explore rehabilitation experiences and specific needs in greater depth. Comparative studies across age groups could provide a clearer understanding of how different demographics experience and benefit from rehabilitation. Additionally, incorporating diverse samples and conducting longitudinal studies would address existing limitations and provide greater clarity on the long-term impacts of rehabilitation on individuals with visual impairments.

## 5. Conclusions

Participants reported that rehabilitation is essential for the development of individuals with visual impairments, highlighting its positive influence on their lives. Additionally, they emphasized that interdisciplinary practices significantly contribute to the performance of daily activities, fostering greater autonomy and independence.

However, while rehabilitation supports school-related demands, the findings indicate a need for effective collaboration among the health, rehabilitation, and education sectors to enhance the teaching and learning processes for individuals with visual impairments and promote their academic development.

Furthermore, participants noted that rehabilitation provides crucial support for families, serving as a safe haven where they can establish strong bonds, feel welcomed, and receive guidance and clarifications needed to secure their rights.

## Data Availability

Data are contained within the article.
